# Supplementary material for: A pairwise cytokine code explains the organism-wide response to sepsis
Source: Nat Immunol. 2024 Jan 8;25(2):226–39. doi: 10.1038/s41590-023-01722-8 (PMC10834370; doi:10.1038/s41590-023-01722-8)
Supplement: Supplementary file 1 — Supplementary Methods. [file 41590_2023_1722_MOESM1_ESM.pdf]

nature immunology

Article https://doi.org/10.1038/s41590-023-01722-8

A pairwise cytokine code explains the organism-wide response to sepsis

In the format provided by the authors and unedited

Supplementary methods

Topic modeling of whole-tissue RNA-seq data

Fitting the topic model

We used fastTopics (version 0.6-142) to fit a topic model to the UMI counts[1,2], with K = 16 topics. fastTopics implements the following two-step approach to fit the topic model: (1) fit a non-negative matrix factorization based on a Poisson model ("Poisson NMF")[3]; (2) recover maximum-likelihood estimates (MLEs) of the topic model parameters by a simple reparameterization[2].

In detail, we took the following steps. First, we removed genes with very low expression (total UMI count ≤ 20). Therefore, the K = 16 topic model was fit to UMI counts for 364 samples and 28,209 genes. Second, we ran 20 expectation maximization (EM) updates, without extrapolation, to get close to an MLE solution ("prefitting phase"). This prefitting phase was implemented in R by calling fit_poisson_nmf from fastTopics with the following settings: numiter = 20, method = "em", init.method = "random", control = list(nc = 8). Third, we performed an additional 180 coordinate descent (CD) updates, with extrapolation, to improve the fit ("refinement phase"). This refinement phase was implemented by calling fit_poisson_nmf with the following settings: method = "scd", numiter = 180, control = list(numiter = 4, nc = 8, extrapolate = TRUE), in which the model fit was initialized using the fit obtained from the prefitting phase. Finally, the topic model was recovered from the Poisson NMF model by calling function poisson2multinom. The convergence diagnostics suggested that, after a total of 200 iterations of the Poisson NMF optimization, the parameter estimates were close to an MLE; the change in log-likelihood between successive iterations was less than 1 × 10-5, and the largest residual in the first order ("Karush-Kuhn-Tucker") conditions was less than 1.

Reassuringly, the estimated topics captured the predominant expression patterns, most of which identify the 13 tissues (the one exception was lymph node and spleen, which shared the same expression pattern). Two other topics (topics 1 and 6) captured variation specific to two tissues (liver and PBMCs), and one topic (topic 9) captured changes in expression over time that were shared across most tissues.

Visualizing the topic proportions

The $n \times K$ matrix of topic proportions, L, where n denotes the number of RNA-seq samples and K is the number of topics, can be viewed as an embedding of the samples in a $(K - 1)$ dimensional space. A simple way to visualize this embedding in 2-d (or 3-d) is to apply a nonlinear dimensionality reduction technique such as *t*-SNE[4] to L. An alternative powerful approach, first suggested by Dey *et al*[1], is to visualize all $K - 1$ dimensions simultaneously using a Structure plot, which has been used with great success in population genetics[5]. The Structure plot is essentially a stacked bar chart, in which bars correspond to samples (rows of L) and bar heights (in different colors, one for each topic) are determined by the topic proportions. To arrange the samples in the Structure plot, we first grouped the RNA-seq samples by tissue, then we ordered them within each tissue by time point.

Differential expression analysis allowing for grades of membership

To annotate the topics, we used the grade-of-membership differential expression (GoM DE) analysis methods[6] and implemented in the de_analysis function in the fastTopics package. In brief, the GoM DE analysis is conceptually similar to a standard DE analysis[7,8], but extends the idea of comparing expression between groups by allowing the cells to have partial membership to multiple

groups (here, the groups are the topics in the topic model). We called the de_analysis function with the following settings: shrink.method = "ash", pseudocount = 0.1 and control = list(ns = 1e5, nc = 8). We performed a second DE analysis, with the same settings, after merging topics 1 and 5 (capturing variation in PBMC expression), 2 and 13 (bone marrow) and topics 6 and 14 (liver). The GoM DE analysis produces, for each gene *j* and topic *k*, estimates of differences in expression, and statistics quantifying support for these differences. In the de_analysis interface, expression differences are defined by the "least extreme" log-fold change ("l.e. LFC"), which is defined for gene *j* and topic *k* as the log-fold change that is the smallest in magnitude among topic pairs (*k*, *l*). After computing initial estimates, the GoM DE analysis (with shrink.method = "ash") performs an adaptive shrinkage step[9], separately for each topic, to stabilize the l.e. LFC estimates. We used the posterior mean estimates, posterior standard errors, posterior z-scores (posterior mean/s.e.) and local false sign rates (*lfsr*) produced by the adaptive shrinkage step to report results of the GoM DE analysis. Note that the *lfsr* can be interpreted similarly to the *q*-value, for example, although the *lfsr* tends to be more conservative than quantities such as the *q*-value that control for the false discovery rate[9].

Gene set enrichment analysis

Mouse gene sets for the gene set enrichment analyses (GSEA) were compiled from the following gene set databases: NCBI BioSystems[10]; Pathway Commons[11,12]; and MSigDB[13–15], which included Gene Ontology (GO) gene sets[16,17]. Specifically, we downloaded bsid2info.gz and biosystems_gene.gz from the NCBI FTP site (https://ftp.ncbi.nih.gov/gene) on March 22, 2020; PathwayCommons12.All.hgnc.gmt.gz from the Pathway Commons website (https://www.pathwaycommons.org) on March 20, 2020; and msigdb_v7.2.xml.gz from the

MSigDB website (https://www.gsea-msigdb.org) on October 15, 2020. For the gene set enrichment analyses, we also downloaded the mouse gene information ("gene info") file Mus_musculus.gene_info.gz from the NCBI FTP site on October 15, 2020. To facilitate integration of these gene sets into our analyses, we have compiled these gene sets into an R package (https://github.com/stephenslab/pathways). We performed two gene set enrichment analyses. In the first GSEA, we included all gene sets other than the following MSigDB collections: C1, C3, C4 and C6, and gene sets labeled as "archived". In the second GSEA, we focused on the curated pathways, specifically gene sets belonging to the GO and CP subcategories in the MSigDB C2 gene set collection. In both analyses, we removed gene sets with fewer than 10 genes and with more than 400 genes. After removing these gene sets, 21,442 candidate gene sets remained for the first analysis, and 8,939 gene sets remained for the second analysis.

We took a simple multiple linear regression approach to the gene set enrichment analysis (GSEA), in which we modeled, for a given topic k, the (least extreme) LFC estimates for all genes using a multiple linear regression model in which the regression variables were gene-set memberships, 1 if gene i belongs to gene set j, otherwise 0. The model fitting for the multiple linear regression model was implemented in SuSiE[18]. The idea behind this simple multiple linear regression approach is that the most relevant gene sets are those that best explain the log-fold changes, and therefore in the multiple regression we sought to identify these gene sets by finding regression coefficients that are nonzero with high probability. Modeling the LFC estimates also helped distinguish among DE genes that show only a slight increase in expression versus those that are highly overexpressed. This simple multiple linear approach ignored uncertainty in the LFC estimates. So, to address this issue, we shrunk the LFC estimates prior to running the GSEA; that is, we defined the regression outcome to be the posterior mean LFC estimate after applying

adaptive shrinkage, as described above. This had the effect that genes that we were more uncertain about had an LFC estimate that was zero or near zero. A benefit to using SuSiE is that it automatically organizes similar or redundant gene sets into credible sets (CSs)[18], making it easier to quickly recognize complementary gene sets.

In detail, the GSEA was performed as follows. We performed a separate GSEA for each topic, Specifically, for topic k, we ran the susieR function susie with the following options: L = 10, intercept = TRUE, standardize = FALSE, estimate_residual_variance = TRUE, refine = FALSE}, compute_univariate_zscore = FALSE and min_abs_corr = 0. We set L = 10 so that SuSiE returned at most 10 credible sets—that is, at most 10 enriched gene sets. For a given topic k, we reported a gene set as being enriched if it was included in at least one CS. We organized the enriched gene sets by credible set (specifically, 95% credible sets). We also recorded the Bayes factor for each CS, which gives a measure of the level of support for that CS. For each gene set included in a CS, we reported the posterior inclusion probability (PIP), and the posterior mean estimate of the regression coefficient. In the results, we refer to the regression coefficient as the enrichment coefficient for a given gene set j since it is an estimate of the expected increase in the LFC for genes that belong to gene set j relative to genes that do not belong to the gene set. Often, a CS contained only one gene set, in which case the PIP for that gene set was close to 1. In several other cases, the CS contained multiple similar gene sets; in these cases, the smaller PIPs indicate that it is difficult to choose among the gene sets because they are similar to each other. (Note that the sum of the PIPs in a 95% CS should always be above 0.95 and less than 1.) Occasionally, SuSiE returned a CS with a small Bayes factor containing a large number of gene sets. When this happened, we did not include these gene sets in the results.

Computing environment for topic modeling and gene set enrichment analyses

Most computations on real data sets were performed in R 3.5.1 (https://www.R-project.org), linked to the OpenBLAS 0.2.19 optimized numerical libraries, on Linux machines (Scientific Linux 7.4) with Intel Xeon E5-2680v4 ("Broadwell") processors. For performing the model topic model optimization and DE analysis, which included multithreaded computations, as many as 8 CPUs and 16 GB of memory were used.

Computing cell type abundance scores from whole-tissue RNA-seq profiles

Database preparation

We used the CellKb database (https://www.cellkb.com), which consists of marker gene sets for mouse cell types. The marker gene sets in CellKb are manually extracted from supplementary materials and raw or processed data from publications describing single-cell or bulk RNA-seq experiments and other publicly available databases, including Tabula Muris (https://tabula-muris.ds.czbiohub.org/), MSigDB (https://www.gsea-msigdb.org/gsea/msigdb/), and the Human Protein Atlas (proteinatlas.org)[14,15,19–21]. Marker gene sets are extensively curated to identify valid genes and cell types. Each marker gene set is associated with the cell type, tissue, organ and/or disease condition given in the source publication, which are mapped to standardized ontology terms using Cell Ontology, Uberon Ontology, and Disease Ontology[22–24]. The quality of the marker gene sets for immune cell types has been evaluated by a comparison with the ImmGen database (https://www.immgen.org/).

Calculation of cell type specificity scores for each gene

A cell type specificity score is calculated for each gene in CellKb based on its specificity and prevalence in marker gene sets of mouse cell types. It defines how specific a gene is for a given cell type and varies between 0 (lowest specificity) and 1 (highest specificity). For this study, we calculated cell type specificity scores for each gene across all mouse cell types using 10,614 marker gene sets extracted from 373 publications as follows:

$$Cell\ type\ specificity\ of\ gene\ i\ in\ cell\ type\ j, c_{ij} = max(0, r_{wt})$$

Where,

- $weighted\ ranked\ of\ gene\ i, r_{wt} = 1 - k\left[log_{10}\left(\frac{r_{min}}{prevalence}\right)\right]$
- $r_{min} = \min(rank\ of\ gene\ i\ in\ all\ marker\ gene\ sets\ of\ cell\ type\ j)$
- $prevalence =$

  $number\ of\ marker\ gene\ sets\ of\ cell\ type\ j\ where\ rank\ of\ gene\ i\ is \leq 500$
- $constant\ k = 0.3$
- $normalized\ cell\ type\ specificity\ of\ gene\ i\ for\ cell\ type\ j, norm\_c_{ij} =$

$$\frac{c_{ij}}{\min\left[1, \max(c_{ij}\ among\ all\ genes\ for\ cell\ type\ j)\right]}$$

Calculation of cell type abundance scores for each cell type in each sample

To identify the cell types whose abundance is changing across tissues and disease conditions, a cell type activity score was calculated for each cell type in each whole-tissue RNA-seq sample as follows:

$$Cell\ type\ activity\ score\ for\ cell\ type\ j\ in\ condition\ k, S_{jk} = \sum_{i=1}^{n}\left(norm\_c_{ij} \times lfc_{ik}\right)$$

Where,

- $norm\_c_{ij} = normalized\ cell\ type\ specificity\ of\ gene\ i\ for\ cell\ type\ j$
- $lfc_{ik} = \log fold\ change\ of\ gene\ i\ in\ condition\ k$
- $normalized\ cell\ type\ activity\ score\ for\ cell\ type\ j\ in\ condition\ k, norm\_S_{jk} = \frac{S_{jk}}{|\max(S_{jk}\ across\ all\ conditions\ m)|}$

Z-scores were calculated for each cell type in each condition across all cell type abundance scores as follows and cell types above an absolute z-score of 1 were considered significant.

$$Zscore\ for\ cell\ type\ activity\ score\ of\ cell\ type\ j\ in\ condition\ k, Z_{jk} = \frac{(S_{jk} - \mu)}{\rho}$$

Where,

- $S_{jk} = cell\ type\ activity\ score\ of\ cell\ type\ j\ in\ condition\ k$
- $\mu = mean\ cell\ type\ activity\ score\ of\ all\ cell\ types\ across\ all\ conditions$
- $\rho = standard\ deviation\ of\ cell\ type\ activity\ score\ of\ all\ cell\ types\ across\ all\ conditions$

References

1. Dey, K. K., Hsiao, C. J. & Stephens, M. Visualizing the structure of RNA-seq expression data using grade of membership models. *PLoS Genet.* **13**, e1006599 (2017).
2. Carbonetto, P., Sarkar, A., Wang, Z. & Stephens, M. Non-negative matrix factorization algorithms greatly improve topic model fits. Preprint at *arXiv* https://doi.org/10.48550/arXiv.2105.13440 (2022).
3. Hien, L. T. K. & Gillis, N. Algorithms for Nonnegative Matrix Factorization with the Kullback–Leibler Divergence. *J. Sci. Comput.* **87**, 93 (2021).

4. Maaten, L. van der & Hinton, G. Visualizing Data using t-SNE. *J. Mach. Learn. Res.* **9**, 2579–2605 (2008).

5. Rosenberg, N. A. *et al.* Genetic Structure of Human Populations. *Science* **298**, 2381–2385 (2002).

6. Carbonetto, P. *et al.* GoM DE: interpreting structure in sequence count data with differential expression analysis allowing for grades of membership. *Genome Biol.* **24,** 236 (2023).

7. Soneson, C. & Robinson, M. D. Bias, robustness and scalability in single-cell differential expression analysis. *Nat. Methods* **15**, 255–261 (2018).

8. Wang, T., Li, B., Nelson, C. E. & Nabavi, S. Comparative analysis of differential gene expression analysis tools for single-cell RNA sequencing data. *BMC Bioinformatics* **20**, 40 (2019).

9. Stephens, M. False discovery rates: a new deal. *Biostatistics* **18**, 275–294 (2017).

10. Geer, L. Y. *et al.* The NCBI BioSystems database. *Nucleic Acids Res.* **38**, D492–D496 (2010).

11. Cerami, E. G. *et al.* Pathway Commons, a web resource for biological pathway data. *Nucleic Acids Res.* **39**, D685–D690 (2011).

12. Rodchenkov, I. *et al.* Pathway Commons 2019 Update: integration, analysis and exploration of pathway data. *Nucleic Acids Res.* **48**, D489–D497 (2020).

13. Subramanian, A. *et al.* Gene set enrichment analysis: A knowledge-based approach for interpreting genome-wide expression profiles. *Proc. Natl. Acad. Sci.* **102**, 15545–15550 (2005).

14. Liberzon, A. *et al.* Molecular signatures database (MSigDB) 3.0. *Bioinformatics* **27**, 1739–1740 (2011).

15. Liberzon, A. *et al.* The Molecular Signatures Database Hallmark Gene Set Collection. *Cell Syst.* **1**, 417–425 (2015).

16. Ashburner, M. *et al.* Gene Ontology: tool for the unification of biology. *Nat. Genet.* **25**, 25–29 (2000).

17. Gene Ontology Consortium. The Gene Ontology resource: enriching a GOld mine. *Nucleic Acids Res.* **49**, D325–D334 (2021).

18. Wang, G., Sarkar, A., Carbonetto, P. & Stephens, M. A simple new approach to variable selection in regression, with application to genetic fine mapping. *J. R. Stat Soc. Series B Stat. Methodol.* **82**, 1273–1300 (2020).

19. Tabula Muris Consortium. A single-cell transcriptomic atlas characterizes ageing tissues in the mouse. *Nature* ***583,*** 590–595 (2020).

20. Tabula Muris Consortium. Single-cell transcriptomics of 20 mouse organs creates a Tabula Muris. *Nature* **562**, 367–372 (2018).

21. Uhlén, M. *et al.* Tissue-based map of the human proteome. *Science* **347**, 1260419 (2015).

22. Diehl, A. D. *et al.* The Cell Ontology 2016: enhanced content, modularization, and ontology interoperability. *J. Biomed. Semantics* **7**, 44 (2016).

23. Mungall, C. J., Torniai, C., Gkoutos, G. V., Lewis, S. E. & Haendel, M. A. Uberon, an integrative multi-species anatomy ontology. *Genome Biol.* **13**, R5 (2012).

24. Schriml, L. M. *et al.* Disease Ontology: a backbone for disease semantic integration. *Nucleic Acids Res.* **40**, D940–D946 (2012).
