# Supplementary material for: A pairwise cytokine code explains the organism-wide response to sepsis
Source: Nat Immunol. 2024 Jan 8;25(2):226–39. doi: 10.1038/s41590-023-01722-8 (PMC10834370; doi:10.1038/s41590-023-01722-8)
Supplement: Supplementary file 2 — Reporting Summary [file 41590_2023_1722_MOESM2_ESM.pdf]

nature portfolio

Corresponding author(s): Nicolas Chevrier

Last updated by author(s): Nov 21, 2023

Reporting Summary

Nature Portfolio wishes to improve the reproducibility of the work that we publish. This form provides structure for consistency and transparency in reporting. For further information on Nature Portfolio policies, see our Editorial Policies and the Editorial Policy Checklist.

Statistics

For all statistical analyses, confirm that the following items are present in the figure legend, table legend, main text, or Methods section.

n/a	Confirmed	
☐	☒	The exact sample size (*n*) for each experimental group/condition, given as a discrete number and unit of measurement
☐	☒	A statement on whether measurements were taken from distinct samples or whether the same sample was measured repeatedly
☐	☒	The statistical test(s) used AND whether they are one- or two-sided<br>*Only common tests should be described solely by name; describe more complex techniques in the Methods section.*
☐	☒	A description of all covariates tested
☐	☒	A description of any assumptions or corrections, such as tests of normality and adjustment for multiple comparisons
☐	☒	A full description of the statistical parameters including central tendency (e.g. means) or other basic estimates (e.g. regression coefficient) AND variation (e.g. standard deviation) or associated estimates of uncertainty (e.g. confidence intervals)
☐	☒	For null hypothesis testing, the test statistic (e.g. *F*, *t*, *r*) with confidence intervals, effect sizes, degrees of freedom and *P* value noted<br>*Give P values as exact values whenever suitable.*
☒	☐	For Bayesian analysis, information on the choice of priors and Markov chain Monte Carlo settings
☐	☒	For hierarchical and complex designs, identification of the appropriate level for tests and full reporting of outcomes
☒	☐	Estimates of effect sizes (e.g. Cohen's *d*, Pearson's *r*), indicating how they were calculated

Our web collection on statistics for biologists contains articles on many of the points above.

Software and code

Policy information about availability of computer code

Data collection	Flow cytometry data and LEGENDplex data were collected with NovoExpress (version 1.3.0) and LEGENDplex software v8. Bulk and spatial RNA-seq data collection and immunohistochemistry image acquisition are detailed in the methods section of the paper.
Data analysis	The following software packages were used for data analysis: Flow cytometry data were analyzed with FlowJo (Version 10.6.2) software. Raw sequencing data were processed using bcbio-nextgen project (version 1.1.5) or STARsolo (version 2.7.10a). Integrated data analysis was performed in R (version 4.2.0 and 3.5.1) or Python (version 3.8.5), based on the packages tidyverse (version 2.0.0), fastTopics (version 0.6-142), data.table (version 1.14.8), cmapR (version 1.8.0), ComplexHeatmap (version 2.12.1), circlize (version 0.4.15), RColorBrewer (version 1.1-3), enrichR (version 3.1), ggrepel (version 0.9.3), patchwork (version 1.1.2), edgeR (version 3.24.3), limma (version 3.38.3), cowplot (version 1.1.0), glue (version 1.4.2), fs (version 1.3.2), Matrix (version 1.2-18), Scanpy (version 1.9.1), scikit-image (version 1.1.3), sckit-learn (version 0.24.2), CARD (version 1.0.0), seaborn (version 0.11.2). ImageJ (version 2.8.0) was used for image analysis. The Space Ranger software was used to process, align, and summarize the FASTQ files. Adobe Illustrator (version 26.2.1) was used for data visualization and figure preparation. Source code is available at 10.5281/zenodo.10158368.

For manuscripts utilizing custom algorithms or software that are central to the research but not yet described in published literature, software must be made available to editors and reviewers. We strongly encourage code deposition in a community repository (e.g. GitHub). See the Nature Portfolio guidelines for submitting code & software for further information.

Data

Policy information about availability of data

All manuscripts must include a data availability statement. This statement should provide the following information, where applicable:

- Accession codes, unique identifiers, or web links for publicly available datasets
- A description of any restrictions on data availability
- For clinical datasets or third party data, please ensure that the statement adheres to our policy

Raw and processed sequencing data are available from the NCBI Gene Expression Omnibus (GEO) repository (accession number: GSE224146).
Source code and preprocessed datasets are available at 10.5281/zenodo.10158368.
We used GRCm39 (GCF_000001635.27) as mouse reference genome.

Research involving human participants, their data, or biological material

Policy information about studies with human participants or human data. See also policy information about sex, gender (identity/presentation), and sexual orientation and race, ethnicity and racism.

Reporting on sex and gender	N/A
Reporting on race, ethnicity, or other socially relevant groupings	N/A
Population characteristics	N/A
Recruitment	N/A
Ethics oversight	N/A

Note that full information on the approval of the study protocol must also be provided in the manuscript.

Field-specific reporting

Please select the one below that is the best fit for your research. If you are not sure, read the appropriate sections before making your selection.

☒ Life sciences ☐ Behavioural & social sciences ☐ Ecological, evolutionary & environmental sciences

For a reference copy of the document with all sections, see nature.com/documents/nr-reporting-summary-flat.pdf

Life sciences study design

All studies must disclose on these points even when the disclosure is negative.

Sample size	No statistical methods were used to predetermine sample sizes, but our sample sizes are similar to those reported in previous publications
Data exclusions	No samples were excluded, unless data acquisition quality was insufficient (e.g., low quality of RNA, or low raw read counts for RNA-seq).
Replication	Biological replicate samples were detailed in the accompanying figure legends or methods. The results were consistent in all independent experiments.
Randomization	No randomization was done as it is not relevant for allocation of mice. Mice were selected according to their genotype, age and sex matched. Mice were randomly assigned for experiments reported. Processing of samples from the various organs did not follow any particular order.
Blinding	Data collection and analysis were not performed blind to the conditions of the experiments.

Reporting for specific materials, systems and methods

We require information from authors about some types of materials, experimental systems and methods used in many studies. Here, indicate whether each material, system or method listed is relevant to your study. If you are not sure if a list item applies to your research, read the appropriate section before selecting a response.

Materials & experimental systems

n/a	Involved in the study	
☐	☒	Antibodies
☒	☐	Eukaryotic cell lines
☒	☐	Palaeontology and archaeology
☐	☒	Animals and other organisms
☒	☐	Clinical data
☒	☐	Dual use research of concern
☒	☐	Plants

Methods

n/a	Involved in the study	
☒	☐	ChIP-seq
☐	☒	Flow cytometry
☒	☐	MRI-based neuroimaging

Antibodies

Antibodies used

CD16/CD32 blocking antibody (clone 93, BioLegend Cat# 101, 1 μL/sample)
https://www.biolegend.com/ja-jp/products/purified-anti-mouse-cd16-32-antibody-190
CD19-FITC (clone 1D3/CD19, BioLegend Cat# 152403, 2 μL/sample)
https://www.biolegend.com/ja-jp/products/fitc-anti-mouse-cd19-antibody-13615
B220-PerCP (clone RA3-6B2, BioLegend Cat# 103233, 4 μL/sample)
https://www.biolegend.com/ja-jp/products/percp-anti-mouse-human-cd45r-b220-antibody-4266
CD93-PE (clone AA4.1, BioLegend Cat# 136503, 4 μL/sample)
https://www.biolegend.com/ja-jp/products/pe-anti-mouse-cd93-aa4-1-early-b-lineage-antibody-6314
CD23-APC (clone B3B4, BioLegend Cat# 101619, 1 μL/sample)
https://www.biolegend.com/ja-jp/products/apc-anti-mouse-cd23-antibody-9762
CD21-Pacific Blue (clone 7E9, BioLegend Cat# 123413, 1 μL/sample)
https://www.biolegend.com/ja-jp/products/pacific-blue-anti-mouse-cd21-cd35-cr2-cr1-antibody-4336
Ter119-FITC (clone TER-119, BioLegend Cat# 116205, 4 μL/sample)
https://www.biolegend.com/ja-jp/products/fitc-anti-mouse-ter-119-erythroid-cells-antibody-1865
CD45-APC-Cy7 (clone 30-F11, BioLegend Cat#103115, 2 μL/sample)
https://www.biolegend.com/ja-jp/products/apc-cyanine7-anti-mouse-cd45-antibody-2530
Zombie Yellow Fixable Viability kit (BioLegend Cat# 423103, 0.5 μL/sample)
https://www.biolegend.com/ja-jp/products/zombie-yellow-fixable-viability-kit-8514
DAPI (Biotium Cat#40043, 1 μL/sample)
https://biotium.com/product/dapi/
anti-TNF (clone XT3.11, BioXCell Cat#BE0058, 50 μg/mouse)
https://bioxcell.com/invivomab-anti-mouse-tnfa?
gad_source=1&gclid=EAIaIQobChMIh_HKo8vUggMVQhJ7Bx3SOwblEAAYASAAEgKCYfD_BwE
anti-IL-18 (clone YIGIF74-1G7, BioXCell Cat#BE0237, 50 μg/mouse)
https://bioxcell.com/invivomab-anti-mouse-il-18-be0237
anti-IFN-γ (clone XMG1.2, BioXCell Cat#BE0055, 50 μg/mouse)
https://bioxcell.com/invivomab-anti-mouse-ifng-be0055
anti-IL-1β (clone B122, BioXCell Cat#BE0246, 50 μg/mouse)
https://bioxcell.com/invivomab-anti-mouse-rat-il-1b
anti-Ly-6G (clone 1A8, BioLegend Cat#127602, 1:100)
https://www.biolegend.com/ja-jp/products/purified-anti-mouse-ly-6g-antibody-4767?GroupID=BLG7232
anti-F4/80 (clone BM8, BioLegend Cat#123102, 1:100)
https://www.biolegend.com/ja-jp/products/purified-anti-mouse-f4-80-antibody-4064

Validation

All antibodies are commercially available and have been validated by manufacturers and in previous publications. The manufacturers' websites provide details regarding validations and associated reference publication (see above section). For flow cytometry, antibodies were titrated in the laboratory before use.

Animals and other research organisms

Policy information about studies involving animals; ARRIVE guidelines recommended for reporting animal research, and Sex and Gender in Research

Laboratory animals

C57BL/6J mice (wild-type, stock 000664), B6.129S7-Ifngtm1Ts/J (Ifng KO, stock 002287), B6.129P2-Il18tm1Aki/J (Il18 KO, stock 004130), C57BL/6J-Il1bem2Lutzy/Mmjax (Il1b KO, stock 068082-JAX), and B6.129S-Tnftm1Gkl/J (Tnf KO, stock 005540) were obtained from the Jackson Laboratories. For all experiments, mice were used at 5-8 weeks of age. Animals were housed in specific pathogen-free and BSL2 conditions at The University of Chicago. Mice were on 12hr light/dark cycles with daylight in Chicago, IL, USA. University of Chicago's Animal facility was maintained at 25 degree c and 30-70% humidity.

Wild animals

The study did not involve wild animals.

Reporting on sex

Only female mice were used for experiments.

Field-collected samples

The study did not involve samples collected from the field.

Ethics oversight	All experiments were performed in accordance with the US National Institutes of Health Guide for the Care and Use of Laboratory Animals and approved by The University of Chicago Institutional Animal Care and Use Committee.

Note that full information on the approval of the study protocol must also be provided in the manuscript.

Flow Cytometry

Plots

Confirm that:

☒ The axis labels state the marker and fluorochrome used (e.g. CD4-FITC).

☒ The axis scales are clearly visible. Include numbers along axes only for bottom left plot of group (a 'group' is an analysis of identical markers).

☒ All plots are contour plots with outliers or pseudocolor plots.

☒ A numerical value for number of cells or percentage (with statistics) is provided.

Methodology

Sample preparation	To analyze splenic B cells, total splenocytes were obtained by mashing spleens on 70-µm filters followed by red blood cell lysis (Lonza). To analysis red blood cell content in the bone marrow, total bone marrow cells were flushed out of femora and tibiae using PBS. Single-cell suspensions were stained in the presence of Fc receptor-blocking antibodies (anti-mouse CD16/32, clone 93) using the following antibodies (BioLegend): CD19-FITC (clone 1D3/CD19, 152403), B220-PerCP (clone RA3-6B2, 103233), CD93-PE (clone AA4.1, 136503), CD23-APC (clone B3B4, 101619), CD21-Pacific Blue (clone 7E9, 123413), Ter119-FITC (clone TER-119, 116205), CD45-APC-Cy7 (clone 30-F11, 103115). Cell viability was measured using Zombie Yellow Fixable Viability kit (423103) or DAPI.
Instrument	Flow Cytometry analysis was performed on a NovoCyte flow cytometer (Acea Biosciences/Agilent)
Software	Flow cytometry data were analyzed with FlowJo (Version 10.6.2) software.
Cell population abundance	Purity was assessed by Flow cytometry analysis. Absolute numbers of cells are outlined in relevant Figures.
Gating strategy	Events were initially gated by FSC-A and SSC-A and then FSC-A and FSC-H were used to exclude doublets. Live cells were gated using a viability dye. Subsequent gating depends on the population of interest and is outlined in Supplementary Information.

☒ Tick this box to confirm that a figure exemplifying the gating strategy is provided in the Supplementary Information.
